# Prevalence, Timing, and Network Localization of Emergent Visual Creativity in Frontotemporal Dementia

**DOI:** 10.1001/jamaneurol.2023.0001

**Published:** 2023-02-27

**Authors:** Adit Friedberg, Lorenzo Pasquini, Ryan Diggs, Erika A. Glaubitz, Lucia Lopez, Ignacio Illán-Gala, Leonardo Iaccarino, Renaud La Joie, Nidhi Mundada, Marguerite Knudtson, Kyra Neylan, Jesse Brown, Isabel Elaine Allen, Katherine P. Rankin, Luke W. Bonham, Jennifer S. Yokoyama, Eliana M. Ramos, Daniel H. Geschwind, Salvatore Spina, Lea T. Grinberg, Zachary A. Miller, Joel H. Kramer, Howard Rosen, Maria Luisa Gorno-Tempini, Gil Rabinovici, William W. Seeley, Bruce L. Miller

**Affiliations:** 1 Department of Neurology, Memory and Aging Center, Weill Institute for Neurosciences, University of California, San Francisco; 2 Neuroscape, University of California, San Francisco; 3 Global Brain Health Institute, University of California, San Francisco, and Trinity College Dublin, Dublin, Ireland; 4 Sant Pau Memory Unit, Department of Neurology, Hospital de la Santa Creu i Sant Pau, Biomedical Research Institute Sant Pau, Universitat Autònoma de Barcelona, Barcelona, Spain; 5 Centro de Investigación en Red-Enfermedades Neurodegenerativas (CIBERNED), Barcelona, Spain; 6 now with Eli Lilly and Company, Philadelphia, Pennsylvania; 7 Department of Radiology and Biomedical Imaging, University of California, San Francisco; 8 Department of Neurology, David Geffen School of Medicine, University of California, Los Angeles; 9 Program in Neurogenetics, Center for Autism Research and Treatment Semel Institute for Neuroscience and Human Behavior, Department of Neurology, David Geffen School of Medicine, University of California, Los Angeles; 10 Department of Human Genetics, David Geffen School of Medicine, University of California, Los Angeles; 11 Institute for Precision Health, University of California, Los Angeles; 12 Department of Pathology, University of California, San Francisco; 13 Associate Editor, *JAMA Neurology*

## Abstract

**Importance:**

The neurological substrates of visual artistic creativity (VAC) are unknown. VAC is demonstrated here to occur early in frontotemporal dementia (FTD), and multimodal neuroimaging is used to generate a novel mechanistic hypothesis involving dorsomedial occipital cortex enhancement. These findings may illuminate a novel mechanism underlying human visual creativity.

**Objective:**

To determine the anatomical and physiological underpinnings of VAC in FTD.

**Design, Setting, and Participants:**

This case-control study analyzed records of 689 patients who met research criteria for an FTD spectrum disorder between 2002 and 2019. Individuals with FTD and emergence of visual artistic creativity (VAC-FTD) were matched to 2 control groups based on demographic and clinical parameters: (1) not visually artistic FTD (NVA-FTD) and (2) healthy controls (HC). Analysis took place between September 2019 to December 2021.

**Main Outcomes and Measures:**

Clinical, neuropsychological, genetic, and neuroimaging data were analyzed to characterize VAC-FTD and compare VAC-FTD with control groups.

**Results:**

Of 689 patients with FTD, 17 (2.5%) met VAC-FTD inclusion criteria (mean [SD] age, 65 [9.7] years; 10 [58.8%] female). NVA-FTD (n = 51; mean [SD] age, 64.8 [7] years; 25 [49.0%] female) and HC (n = 51; mean [SD] age, 64.5 [7.2] years; 25 [49%] female) groups were well matched to VAC-FTD demographically. Emergence of VAC occurred around the time of onset of symptoms and was disproportionately seen in patients with temporal lobe predominant degeneration (8 of 17 [47.1%]). Atrophy network mapping identified a dorsomedial occipital region whose activity inversely correlated, in healthy brains, with activity in regions found within the patient-specific atrophy patterns in VAC-FTD (17 of 17) and NVA-FTD (45 of 51 [88.2%]). Structural covariance analysis revealed that the volume of this dorsal occipital region was strongly correlated in VAC-FTD, but not in NVA-FTD or HC, with a volume in the primary motor cortex corresponding to the right-hand representation.

**Conclusions and Relevance:**

This study generated a novel hypothesis about the mechanisms underlying the emergence of VAC in FTD. These findings suggest that early lesion-induced activation of dorsal visual association areas may predispose some patients to the emergence of VAC under certain environmental or genetic conditions. This work sets the stage for further exploration of enhanced capacities arising early in the course of neurodegeneration.

## Key Points

**Question:**

What are the neural underpinnings of visual artistic creativity (VAC) in the setting of frontotemporal dementia (FTD)?

**Finding:**

In this study of 119 patients, VAC occurred early in the course of FTD, was disproportionately observed in patients with temporal lobe–predominant degeneration, and may be associated with damage to brain regions that normally suppress dorsomedial occipital cortex function.

**Meaning:**

This work generated a novel hypothesis about the mechanisms underlying the emergence of VAC in FTD, setting the stage to study in-depth enhanced capacities arising early in the course of neurodegeneration.

## Teaser

This case-control study assesses the anatomical and physiological underpinnings of visual artistic creativity in individuals with frontotemporal dementia.

## Introduction

Creativity, the ability to generate work that is both novel and valuable,^1^ is pivotal to the development of human culture, as it enables transformative problem solving, technological progress, and artistic expression. Visual artistic creativity (VAC) is defined as the production of novel and aesthetically pleasing visual forms and is a process that depends heavily on visual mental imagery.^2^ VAC is unique to and ubiquitous in human societies,^3^ and insights into VAC have the potential to illuminate the neural underpinnings of creativity more broadly.^4,5,6,7,8^ Frontotemporal dementia (FTD) represents a group of neurodegenerative disorders characterized by progressive deterioration of behavior and/or language, usually associated with focal frontotemporal lobar degeneration pathology affecting the frontal, insular, and temporal cortex. Emergence of novel visual artistic skills has been described in FTD, particularly in the primary progressive aphasia (PPA) variants,^9,10,11,12^ which result from degeneration of the left frontal and anterior temporal lobes. Moreover, patients with focal brain lesions caused by stroke and traumatic brain injury have been reported to develop new visual artistic skills,^13,14,15^ most often after injury to brain regions affected in FTD. Therefore, patients with anterior brain lesions provide a rare window into the neural network building blocks of VAC. Previously, we speculated that selective degeneration of the frontal and anterior temporal lobes, within the language-dominant hemisphere, led to decreased inhibition of posterior visual-spatial systems involved in visual perception and association, thereby enhancing artistic interest.^12^ This hypothesis has not been systematically examined, and the underlying neural mechanisms for VAC in the setting of brain injury remain uncertain.

We describe the clinical, neuropsychological, neuropathological, and genetic features of 17 patients with FTD, drawn from a cohort of 734 patients assessed over 17 years, who reported emergence of VAC. We then probe the neural substrates of this phenomenon using atrophy network mapping and structural covariance analyses, applied to patients with FTD with and without VAC as well as healthy controls.

## Methods

### Participants

Patients were evaluated at the University of California, San Francisco, Memory and Aging Center as part of a prospective, longitudinal cohort study focused on FTD spectrum disorders. Visits occurred between January 2002 and May 2019. Analysis took place between September 2019 and December 2021. All participants provided written informed consent, and the University of California, San Francisco, Committee on Human Research approved the study. Patients underwent standardized clinical, neuropsychological, and neuroimaging evaluations.^16^ Genotyping for autosomal dominant pathogenic genetic variants in *MAPT*, *GRN*, and *C9orf72* was performed as previously described.^17^ Inclusion criteria included a clinical diagnosis of behavioral variant of FTD, nonfluent variant of PPA, semantic variant of PPA (svPPA), progressive supranuclear palsy with Richardson syndrome, corticobasal syndrome, or amyotrophic lateral sclerosis, diagnosed according to the prevailing clinical research criteria at the time of assessment.^18,19,20,21,22,23,24,25^ These criteria yielded a pool of 734 patients, from which 45 were excluded due to lack of available clinical records. For the remaining 689 patients, retrospective medical record review was performed by a single investigator (A.F., a behavioral neurologist) to identify patients who met the following criteria for emergent VAC: (1) emergence of novel visual artistic skills, (2) a substantial increase in quantity of visual art generated, or (3) a change in the style of visual art produced. Participant inclusion based on criterion 3 alone required that the change of artistic style was not attributable to semantic loss (eMethods 1 in Supplement 1). To ensure that the ascertainment strategy was reproducible, we conducted an interrater reliability analysis on 68 participants with 2 additional raters (M.K. and K.N.), each blinded to the initial assessment by the first investigator (A.F.). This analysis showed high rates of agreement (Fleiss κ evaluating agreement among all 3 raters was 0.84; eMethods 2 in Supplement 1). Demographic features and description of visual artistic behavior were recorded, and all available artwork was collected. Data on race and ethnicity were self-reported. To evaluate the neural signature unique to patients with emergence of VAC in the setting of FTD, 2 control groups were assembled: (1) not visually artistic FTD (NVA-FTD) and (2) healthy controls (HC). NVA-FTD included patients with FTD spectrum disorders for whom no change in visual artistic behavior was reported. Patients with NVA-FTD were matched to patients with an FTD spectrum disease and emergence of visual artistic creativity (VAC-FTD) by clinical diagnosis, disease stage (assessed using the Clinical Dementia Rating Scale sum of boxes score),^26^ age, sex, handedness, and years of education. A matching process accounting for multiple variables was required, and we matched 3 controls to each VAC-FTD participant. The HC group was matched for age, sex, handedness, and years of education. Between-group differences in clinical and neuropsychological characteristics were assessed using *t* test, Mann-Whitney test, analysis of variance, or Kruskal-Wallis test for continuous variables and χ^2^ and Fisher exact test for categorical data, as appropriate. Test statistics were considered significant at *P* < .05 (2-tailed). Statistical analyses were performed with R version 4.1.1 (R Foundation for Statistical Computing; eMethods 3 in Supplement 1). Strengthening the Reporting of Observational Studies in Epidemiology (STROBE) reporting guideline was followed in preparing this report.

### Structural Magnetic Resonance Imaging Acquisition and Preprocessing

Over the ascertainment period, magnetic resonance images were acquired with 4 different scanners using several image acquisition protocols (eTable 1 and eMethods 4 in Supplement 1). Magnetic field strength was 1.5, 3.0, or 4.0 T. Structural images were preprocessed using voxel-based morphometry (eMethods 5 in Supplement 1).^27^

### Individual Atrophy (W-Score) Maps

To generate participant-specific atrophy maps, the smoothed gray matter images were transformed into W-score maps (eMethods 6 in Supplement 1). W-score maps are voxelwise statistical maps that reflect levels of atrophy for each individual after adjustment for relevant covariates.^28,29^ The W-score model generated for this study was based on 397 HCs and included age at magnetic resonance imaging, sex, years of education, handedness, scanner type, and total intracranial volume as covariates.

Threshold individual W-score maps were calculated and binarized to capture each patient’s 1% most atrophied voxels. This procedure enabled us to represent patients’ focal neurodegeneration in a balanced manner across patients of varying overall atrophy severity and extent. To ensure robustness of findings across a range of atrophy thresholds, we repeated all analyses with more (highest, 0.5%) and less (highest, 2%-5%) stringent thresholds.

### Comparing W-Score Maps in VAC-FTD vs NVA-FTD

We compared the unthresholded W-score maps between groups (VAC-FTD vs NVA-FTD) at every voxel using a 2-sample *t* test. Mini-Mental State Examination (MMSE)^30^ score was included as a nuisance covariate. Significant clusters were identified, across the whole brain, using a *t* threshold corresponding to *P* < .001 uncorrected for multiple comparisons.

### Atrophy Network Mapping

Next, we sought to determine how the brain areas atrophied in patients are functionally connected in the healthy brain to other brain regions. First, we derived an atrophy network map for each patient, seeded by each patient’s binarized single-patient atrophy map (top 1% most atrophied voxels).^31,32,33,34^ Using this seed region, we turned to task-free functional magnetic resonance imaging data from a cohort of 175 cognitively healthy individuals, matched to the VAC-FTD group by age, sex, handedness, and years of education (eTable 2 in Supplement 1). In these patients, we computed the mean blood oxygen level–dependent signal time series for all voxels within the patient-derived atrophy seed region and correlated these mean time series with the time series of every other voxel (eMethods 7 in Supplement 1). Resulting *r* values were converted to a normal distribution using Fisher *r*-to-*z* transform and entered into a single, group-level, voxelwise 1-sample *t* test. The resulting maps constituted the unthresholded atrophy network *t* maps. Positive and negative functional correlations were thresholded at *t *≥* *|7|, corresponding to *P* < 10^−10^, to create a binarized map of connected regions, in keeping with prior approaches.^35^ To ensure that results were not dependent on this threshold, we repeated our analysis with *t *value thresholds of 6 (*P* < 10^−7^) and 8 (*P* < 10^−12^). Finally, the binarized atrophy network maps were overlaid to generate frequency maps for each patient group, representing the proportion of patients in that group whose lesions were functionally connected to each voxel in the healthy brain (eFigure 1 in Supplement 1). We also compared the unthresholded atrophy network *t* maps between the VAC-FTD and NVA-FTD groups, voxelwise, using a 2-sample *t* test. Significant clusters were identified across the whole brain using a *t* threshold corresponding to *P* < .001, uncorrected for multiple comparisons.

### Structural Covariance Analysis

Atrophy network mapping results were used as the basis for investigating differences in interregional structural correlations between VAC-FTD and NVA-FTD.^36,37^ To that end, a voxelwise interaction model was implemented in SPM12 (Wellcome Centre for Human Neuroimaging, UCL Queen Square Institute of Neurology) using the W-score maps of the VAC-FTD and NVA-FTD groups and adding a term for the individual mean W-score of the region of interest (ROI) as a covariate. An interaction term between group membership (VAC-FTD/NVA-FTD) and individual mean W-score of the ROI was included, and a statistical contrast was set to elicit group differences in covariance between the individual mean W-score of the ROI and other brain regions, with MMSE score included as a confounding covariate. A second interaction model examined group differences in structural covariance to the ROI in individuals with VAC-FTD vs HCs. In this model, MMSE was omitted because it is highly confounded with group. A statistical threshold of *P* < .001 (whole brain, uncorrected) with a minimum cluster extent of 100 voxels and cluster-level threshold of *P* < .05 (uncorrected) was applied for both interaction models. To examine the differential structural covariance of the ROI with multiple cortical brain regions, we calculated the mean W-score for each cortical Brainnetome atlas^38^ parcel and computed its correlation to the mean W-score of the ROI. We repeated this for the VAC-FTD, NVA-FTD, and HC groups and used Kruskal-Wallis test to compare the resulting distributions.

### ^18^F-Fluorodeoxyglucose Positron Emission Tomography

^18^F-fluorodeoxyglucose positron emission tomography (FDG-PET) images were acquired and preprocessed using standardized methods (eMethods 8 in Supplement 1). Based on standardized uptake value ratio (SUVR) maps of 71 HCs, a voxelwise FDG-PET W-score model was generated including age, sex, handedness, and education as covariates. For a single patient who had FDG-PET scanning 14 months apart, before and after emergence of VAC, individual voxelwise FDG-PET W-score maps were generated. For these 2 maps, mean regional SUVR W-scores were extracted from Brainnetome atlas parcels^38^ (eMethods 9 in Supplement 1).

## Results

### Clinical and Demographic Characteristics

**Figure 1.**
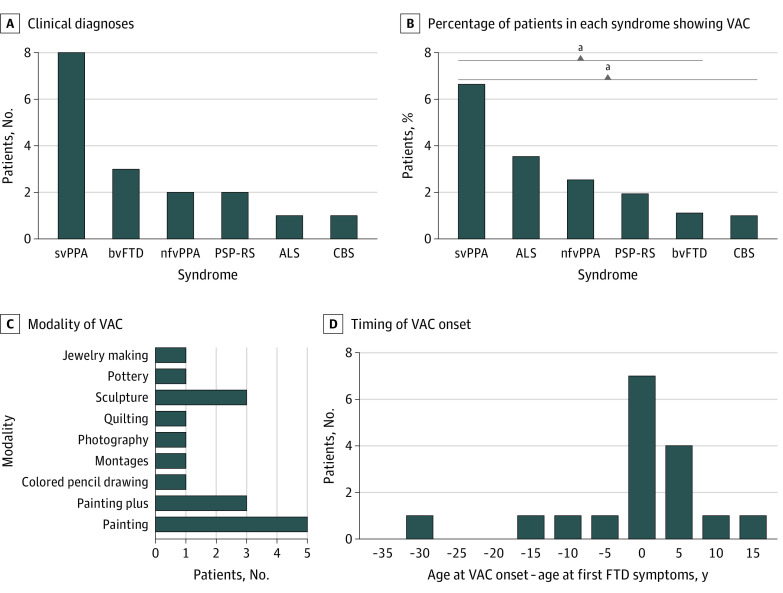
Clinical Characteristics and Artistic Expression in Patients With Frontotemporal Dementia (FTD) Who Reported Emergence of Visual Artistic Creativity (VAC) (n = 17) A, Semantic variant of primary progressive aphasia (svPPA) was the most common clinical syndrome in the VAC-FTD group. B, SvPPA was also the FTD syndrome with the highest percentage of patients with VAC relative to the overall syndrome-based cohort, significantly higher than in behavioral variant of frontotemporal dementia (bvFTD) and corticobasal syndrome (CBS) (Fisher exact test, *P* < .05). C, Painting was the most common modality of visual artistic expression. Painting plus indicates that painting, the primary modality, was accompanied by other forms of visual art: sculpture (n = 1), photography (n = 1), jewelry making, and glass painting (n = 1). D, Most patients showed emergence of visual artistic creativity in close temporal proximity to FTD symptom onset. ALS indicates amyotrophic lateral sclerosis; nfvPPA, nonfluent variant of primary progressive aphasia; PSP-RS, progressive supranuclear palsy with Richardson syndrome. ^a^*P* < .05 for svPPA vs bvFTD and for svPPA vs CBS.

Of 689 patients with FTD, 17 (2.5%) met VAC-FTD inclusion criteria (mean [SD] age, 65 [9.7] years; 10 [58.8%] female). NVA-FTD (n = 51; mean [SD] age, 64.8 [7] years; 25 [49.0%] female) and HC (n = 51; mean [SD] age, 64.5 [7.2] years; 25 [49%] female) groups were well matched to VAC-FTD demographically. Overall, 17 met the operational definition of emergent VAC, with a resulting prevalence of 2.5%. Eight of 17 patients had de novo emergence of VAC, 7 showed some past interest in either visual or nonvisual art, and 2 were artists who experienced substantial change in artistic style. The most frequently associated FTD clinical syndrome was svPPA, accounting for nearly half of the cases (8 of 17 [47%]) and occurring in 6.7% of all patients with svPPA (8 of 120) (Figure 1 and eTable 3 in Supplement 1). No pathogenic variants in *C9orf72*, *GRN,* or *MAPT* were found in the 15 of 17 patients (88.2%) with VAC-FTD who underwent genetic testing. Neuropathological diagnoses available for 6 of 17 patients (35.3%) with VAC-FTD revealed diverse underlying focal frontotemporal lobar degeneration subtypes (eTable 4 in Supplement 1).

Emergence of VAC occurred early in the FTD disease course. Most patients experienced the change at, or shortly after, the time of FTD symptom onset, but 4 of 17 patients (32.5%) showed emergent VAC before their FTD symptoms appeared (Figure 1). As mandated by the study design, individuals with VAC-FTD and NVA-FTD showed no significant differences in demographic or clinical variables. MMSE score was higher in the VAC-FTD group than the NVA-FTD group, reflecting better preserved memory in this group (Table). Consequently, MMSE was used as a nuisance covariate in neuroimaging analyses. Prevalence of neuropsychiatric symptoms measured by the Neuropsychiatric Inventory was similar in the 2 groups.^39^

**Table.** Participants’ Demographic and Neuropsychological Features^a^

Characteristic	Mean (SD)			*P* value
	VAC-FTD (n = 17)	NVA-FTD (n = 51)	HC (n = 51)	
Clinical diagnosis, No. (%)				
svPPA	8 (47.1)	24 (47.1)	NA	NA
bvFTD	3 (17.6)	9 (17.6)	NA	NA
nfvPPA	2 (11.8)	6 (11.8)	NA	NA
PSP-RS	2 (11.8)	6 (11.8)	NA	NA
CBS	1 (5.9)	3 (5.9)	NA	NA
ALS	1 (5.9)	3 (5.9)	NA	NA
Age at MRI scan, y	65 (9.7)	64.8 (7)	64.5 (7.2)	>.99
Sex, No. (%)				
Male	7 (41.1)	26 (51.0)	26 (51.0)	.76
Female	10 (58.8)	25 (49.0)	25 (49.0)	
Handedness, No. (%)				
Right	14 (82.4)	42 (82.4)	43 (84.3)	.96
Left	3 (17.6)	9 (17.6)	8 (15.7)	
Education, y^b^	16.1 (4.7)	16.6 (2.1)	16.7 (2)	.77
Race and ethnicity, No. (%)				
Asian	0	1 (2.0)	8 (15.7)	NA
Hispanic	0	1 (2.0)	0	
White	17 (100)	49 (96.1)	38 (74.5)	
Not reported	0	0	5 (9.8)	
CDR-SB (maximum, 18)	4.6 (2.8)	4.6 (3.1)	0 (0)	.94
Mini-Mental State Examination score (maximum, 30)^c^	27.1 (2.6)	22.7 (6.2)	NA	.004
Boston Naming Test (maximum, 15)^c^	10.4 (4.5)	8.4 (5.5)	NA	.17
Phonemic fluency (D words/1 min)^c^	7.4 (4.2)	7.3 (4.8)	NA	.52
Semantic fluency (animals/1 min)	10.9 (4.4)	9.8 (6)	NA	.46
Modified Rey-O copy (maximum, 17)^c^	15.3 (1.2)	14.6 (2.5)	NA	.53
CVLT SF				
4 Trials (maximum, 36)	22.1 (5.3)	17.6 (7.7)	NA	.01
30-s Recall (maximum, 9)	5.6 (2)	3.8 (2.8)	NA	.01
10-min Recall (maximum, 9)^c^	4.4 (2.9)	3.1 (3)	NA	.14
Recognition hits^c^	7.4 (1.5)	7.1 (2.3)	NA	>.99
Modified Rey-O Figure				
10-min Delay (maximum, 17)	9.9 (3.4)	6.4 (4.7)	NA	.002
Recognition (yes/no)	0.8 (0.4)	0.8 (0.4)	NA	.72
Digit span backward^c^	4.4 (1.2)	4.1 (1.2)	NA	.35
Modified trails				
Correct lines/1 min	22.7 (15.4)	19.3 (12.3)	NA	.42
Errors^c^	1.1 (1.4)	1 (1.3)	NA	.81
Design fluency (correct designs/1 min)	8.2 (3.7)	6.5 (3.1)	NA	.14
NPI subscale responses, yes, No. (%)				
Delusions	2 (12)	5 (10)	NA	.82
Hallucinations	1 (6)	3 (6)	NA	>.99
Agitation	8 (47)	22 (43)	NA	.78
Elation	3 (18)	22 (43)	NA	.06
Anxiety	8 (47)	23 (45)	NA	.89
Depression	6 (35)	24 (47)	NA	.40
Irritability	8 (47)	25 (49)	NA	.89
Motor disturbance	8 (47)	31 (61)	NA	.32
Nighttime behaviors	6 (35)	31 (61)	NA	.07
Appetite	10 (59)	35 (69)	NA	.46
Apathy	10 (59)	38 (75)	NA	.22
Disinhibition	9 (53)	38 (75)	NA	.10

Abbreviations: ALS, amyotrophic lateral sclerosis; bvFTD, behavioral variant of frontotemporal dementia; CBS, corticobasal syndrome; CDR-SB, Clinical Dementia Rating scale sum of boxes; CVLT SF, California Verbal Learning Test Short Form; HC, healthy controls; MRI, magnetic resonance image; NA, not applicable; nfvPPA, nonfluent variant of primary progressive aphasia; NPI, Neuropsychiatric Inventory; NVA-FTD, frontotemporal dementia spectrum disease without emergence of visual artistic creativity; PSP-RS progressive supranuclear palsy with Richardson syndrome; svPPA, semantic variant of primary progressive aphasia; VAC-FTD, frontotemporal dementia spectrum disease and emergence of visual artistic creativity.

^a^ All cognitive measures were available for at least 80% of participants in each group. NPI measures were available for all participants.

^b^ Kruskal-Wallis test was used.

^c^ Mann-Whitney test was used.

### Visual Art

**Figure 2.**
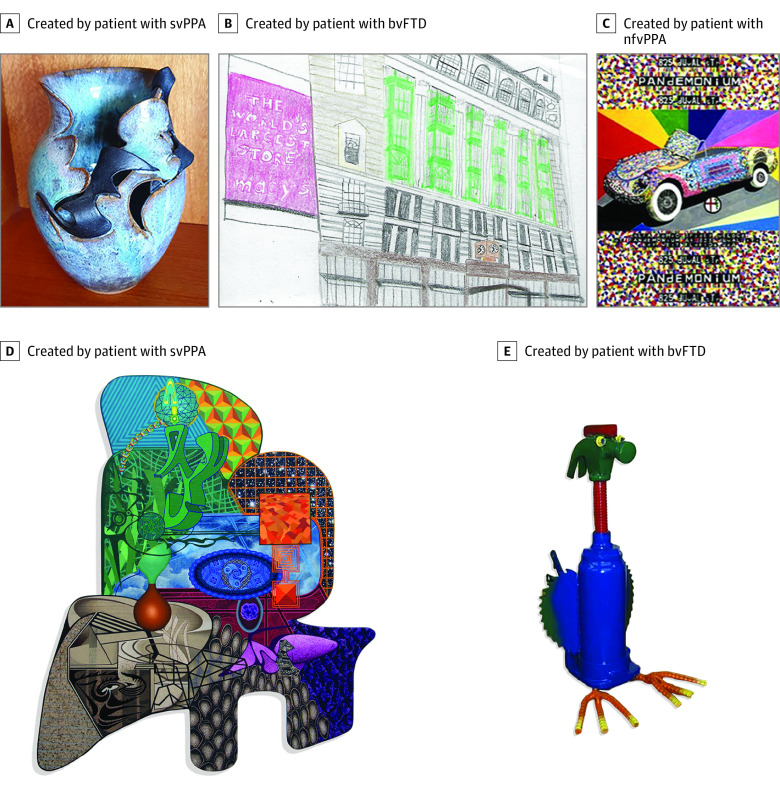
Examples of the Visual Artworks Each piece was selected to represent the style of a single patient with a frontotemporal dementia spectrum disease and emergence of visual artistic creativity. Artworks presented were generated by patients with clinical diagnoses of semantic variant of primary progressive aphasia (svPPA) (A and D), behavioral variant of frontotemporal dementia (bvFTD) (B and E), and nonfluent variant of primary progressive aphasia (nfvPPA) (C).

Visual art collected from 11 of 17 patients (64.7%) included painting, quilting, jewelry making, sculpture, pottery, and montage making (eTable 3 in Supplement 2). Bright colors were common, and the art rarely focused on human faces. In some, there was evidence for loss of semantic knowledge. For example, 2 patients with svPPA generated animal sculptures lacking the features of a species, producing generic or prototypical representations of an animal. When humans and animals were depicted, facial expressions were often bizarre and did not convey natural emotions, as has been previously described (Figure 2).^40,41^

### Structural Magnetic Resonance Imaging

Patients in the VAC-FTD and NVA-FTD groups showed typical group-level atrophy maps highlighting neurodegeneration in the anterior temporal lobes (left greater than right), amygdalae, striatum, and left insula (eFigures 2 and 3 in Supplement 1). Statistical W-score map and gray matter map comparisons between the VAC-FTD and NVA-FTD groups revealed no group differences.

### Atrophy Network Mapping

**Figure 3.**
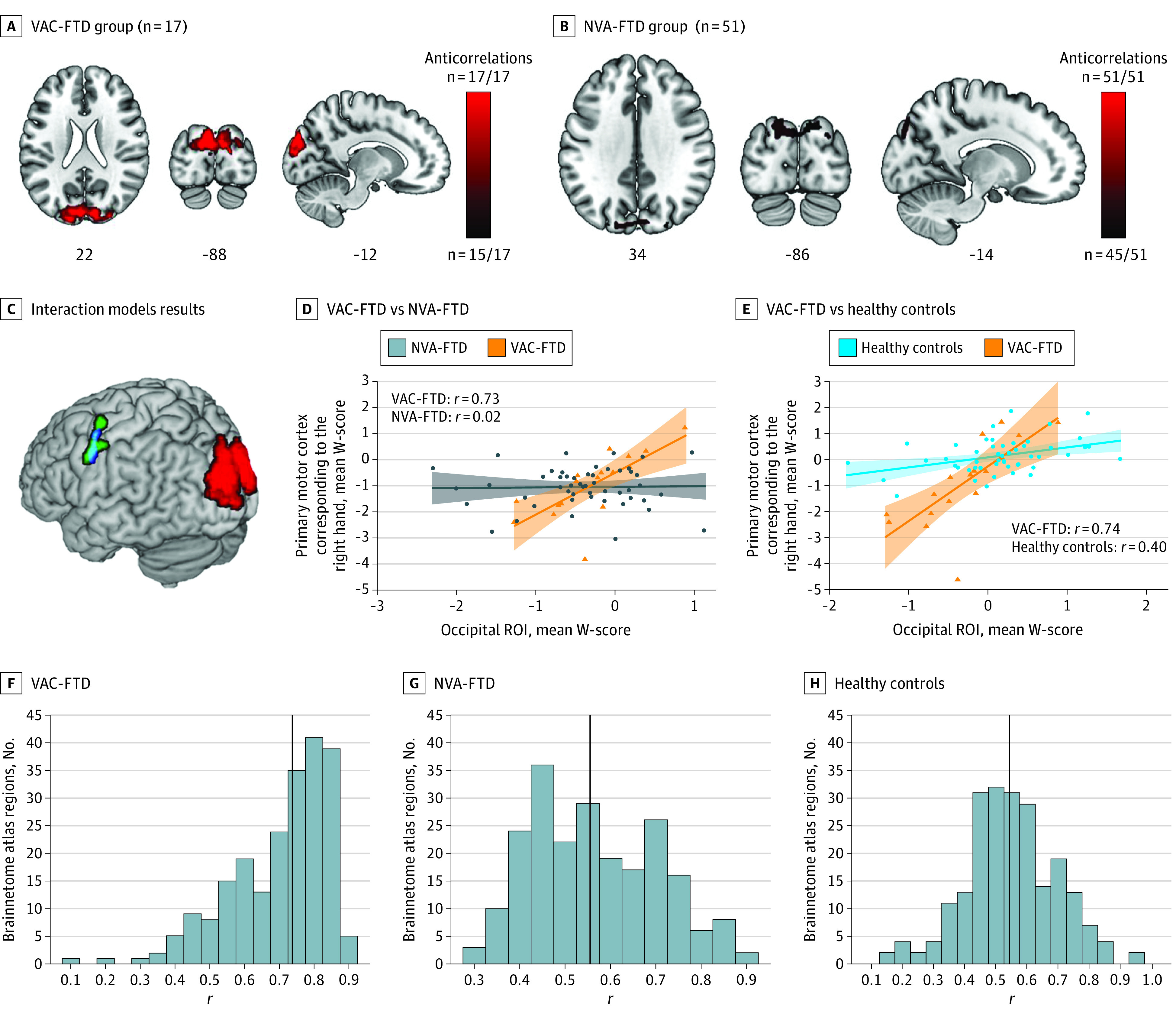
Atrophy Network Mapping and Structural Covariance Analyses Results A and B, Individual atrophy network maps were thresholded at *t* ≥ |7|, binarized, and overlaid to create group-level atrophy network maps. A similar bilateral dorsomedial occipital region showed anticorrelated brain activity, in healthy controls, to the activity seen in the top 1% of atrophied voxels in both individuals with visual artistic creativity with frontotemporal dementia (VAC-FTD) (17 of 17 patients) and patients with a frontotemporal dementia spectrum disease without emergence of visual artistic creativity (NVA-FTD) (45 of 51 patients [88.2%]). C-E, An interaction model, designed to detect brain regions showing greater structural covariance with the dorsomedial occipital region (red) in VAC-FTD vs NVA-FTD, identified a cluster in the right-hand region of the primary motor cortex (green). This cluster was positively correlated with the mean W-score in the occipital region of interest (ROI) in VAC-FTD but showed no correlation in NVA-FTD (D). A similar cluster (blue, panel C) was observed in a second interaction model aimed to identify regions with greater positive correlation in VAC-FTD than in HC (data shown in E). F-H, Distributions of the correlations of the mean W-score of the dorsomedial occipital ROI and the mean W-score of cortical brain regions (parcellated by the Brainnetome atlas) are shown. The VAC-FTD group had significantly stronger positive structural correlations with the ROI. Findings in panels A-C are superimposed on slice and render images of the Montreal Neurological Institute template brain. Images are in neurological orientation (left = left). D and E, The shaded areas represent the 95% CIs for the fitted regression lines. F-H, The vertical black line represents the median.

Threshold individual atrophy network maps were calculated at t ≥ |7|, binarized, and overlaid to generate a group level atrophy network frequency map for the VAC-FTD and NVA-FTD groups. Group-level atrophy network frequency maps identified a bilateral dorsomedial occipital region anticorrelated in the healthy brain to the atrophy patterns of 17 of 17 participants with VAC-FTD. A similar but smaller cluster was revealed in 45 of 51 participants with NVA-FTD (88.2%) (Figure 3). No brain regions positively correlated with the top 1% of atrophied voxels were detected using these thresholds. These findings were reproduced across a range of atrophy thresholds and individual lesion network map thresholds t ≥ 6 and t ≥ 8 (eFigures 4 and 5 in Supplement 1). The occipital cluster was consistently more extensive in the VAC-FTD vs NVA-FTD groups at the same threshold, but a voxelwise 2-sample *t* test comparing unthresholded *t* maps from VAC-FTD and NVA-FTD found no group differences. A secondary analysis comparing only patients with svPPA from both groups was also unrevealing.

### Structural Covariance Analysis

Having identified a dorsomedial occipital region that anticorrelated with the regions atrophied in FTD and was also more prominent in individuals with VAC-FTD, we hypothesized that this region would show unique structural covariation patterns in individuals with VAC-FTD vs NVA-FTD (eMethods 10 in Supplement 1).

Structural covariance mapping interrogates neural systems by leveraging between-patient correlations in gray matter volume across the brain.^36,37^ To test our hypothesis, we used the dorsomedial occipital ROI to investigate group differences in interregional structural covariance, which may result from long-standing large-scale functional coupling alterations. Our interaction model revealed 2 clusters significantly correlated with the ROI in VAC-FTD but not in NVA-FTD. The first cluster localized to the left primary motor cortex, in the neighborhood of the motor representation of the right hand (−54; −2; *z* = 39; *k* = 437 voxels; cluster-level *P* = .01). A second significant cluster was detected in the superior temporal gyrus (58; −39; *z* = 15; *k* = 361 voxels; cluster-level *P* = .02). No region showed greater structural covariance in NVA-FTD than in VAC-FTD, and no region showed greater gray matter anticorrelation with the seed in either contrast.

To ensure that these group differences were not driven by the MMSE differences, we assembled an additional FTD control group matched also for MMSE (n = 34). No statistically significant correlation was found between the seed and the right-hand cluster in this better-matched NVA-FTD group (eTable 5 and eFigure 6 in Supplement 1). We then addressed whether these structural associations were also greater in individuals with VAC-FTD than in HCs. The second interaction model identified an overlapping cluster in the primary motor cortex (−53; −3; *z* = 41; *k* = 183 voxels; cluster-level *P* = .045) region but not in the superior temporal gyrus (eTable 6 in Supplement 1). Examining the differential structural covariance of cortical brain regions with the dorsomedial occipital ROI revealed that those with VAC-FTD had significantly stronger positive structural correlations (median [IQR] Pearson *r*, 0.74 [0.61-0.81]) than those with NVA-FTD (0.55 [0.45-0.68]) and HCs (0.54 [0.46-0.65]) (*P* < .001).

### Single Case With Longitudinal FDG-PET

**Figure 4.**
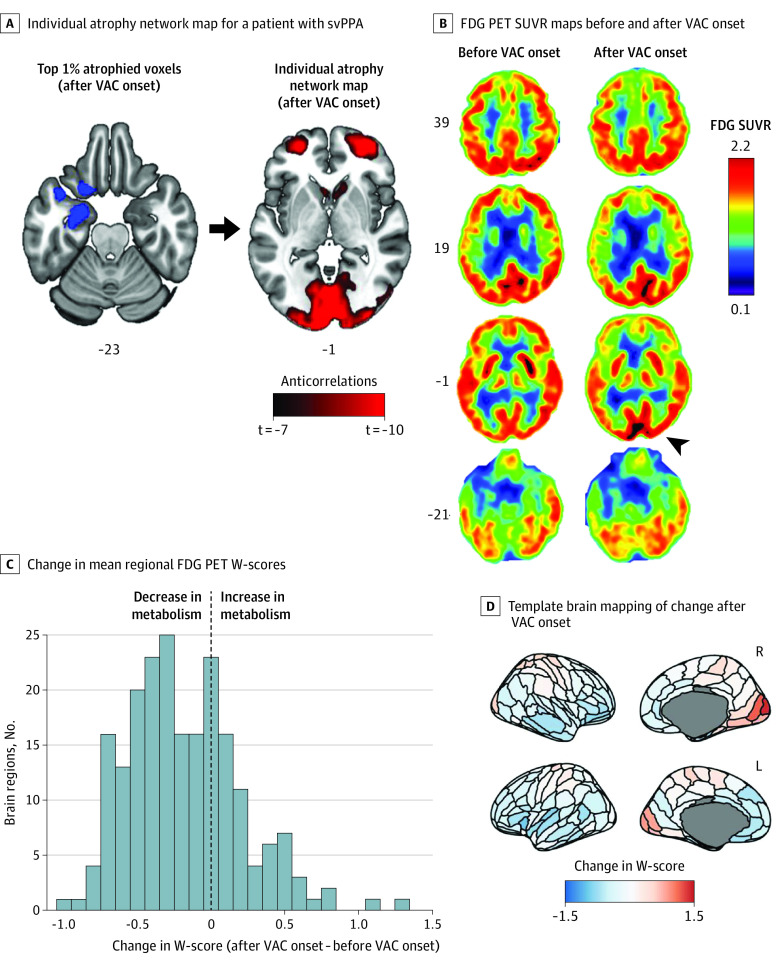
Atrophy Network Mapping and Intensified Occipital Glucose Metabolism After Emergence of Visual Artistic Creativity (VAC) in a Patient With Semantic Variant of Primary Progressive Aphasia (svPPA) A, Top 1% most atrophied voxels from a patient with svPPA and emergent VAC were used as seed to obtain an individual atrophy network map based on the healthy brain connectome. The left anterior temporal lobe regions atrophied in the patient (blue) showed anticorrelated brain activity, in controls, to medial occipital regions (red). B, ^18^F-fluorodeoxyglucose positron emission tomography (FDG-PET) standardized uptake value ratio (SUVR) maps obtained before and after emergence of VAC show decline in glucose metabolism in temporal and frontal regions bilaterally, accompanied by increasing metabolism in medial occipital regions, right greater than left (arrowhead). C and D, Mean regional FDG-PET W-scores were computed for individual brain regions across the whole brain. For each region, the W-score after VAC onset was subtracted from that obtained prior to VAC onset to generate a corresponding change value for each region. Multiple brain regions, most prominently primary visual and visual association areas and sensorimotor regions, showed increased metabolism after VAC onset, as mapped on a template brain in panel D. Images are in neurological view (left = left).

The cross-sectional analyses presented above suggest that dorsal occipital structure or function becomes enhanced early in FTD, sometimes in association with the appearance of VAC. Remarkably, 1 patient in the VAC-FTD group, with a clinical diagnosis of svPPA, underwent FDG-PET scanning 14 months apart, before and after she started painting. This patient did not pursue painting until after word-finding difficulties emerged and used colored pencils, preferring bright colors and nonhuman subjects. Leveraging this rare opportunity, we assessed changes in glucose metabolism around the time their creativity blossomed. FDG-PET images obtained before and after VAC onset showed metabolic decline in anterior temporal and frontal regions, as expected, but preserved or increased metabolism in numerous posterior regions (Figure 4A and B). To quantify these changes across the brain, for each region, mean SUVR W-score from the first scan was subtracted from the second scan to produce a regional change map (Figure 4C and D). Ten brain regions, most in the occipital lobes, showed increased metabolism by more than 0.5 W-score units (eTable 7 in Supplement 1). Mean regional SUVR W-scores of the dorsomedial occipital ROI uncovered by atrophy network mapping revealed an increase in FDG-PET W-score in parallel to emergence of VAC (first scan: W = 0.00, second scan: W = 0.83; eFigure 7 in Supplement 1). Matched controls with svPPA and longitudinal FDG-PET data were not available to examine the specificity of the increase in metabolism to patients with FTD with emergence of VAC.

## Discussion

Emergence of VAC in FTD occurs in 2.5% of patients and is disproportionately associated with svPPA (6.7%). Remarkably, this VAC occurs in the setting of neurodegeneration and results in distinct forms of visual artistic expression. VAC emerges early in the disease course, around the time of FTD symptom onset, as supported by a recent review of single cases reported in the literature.^11^

Atrophy network mapping enables researchers to pinpoint network nodes commonly connected in the healthy brain to lesions from a neurodegenerative disease group of interest. The method is well suited to uncovering the network basis for aberrant gains of function,^32,33,34^ such as the emergence of VAC studied here. We found that the varied regions of peak frontotemporal atrophy across patients were united by a functional activity pattern that inversely correlated with dorsomedial occipital cortex. This region, encompassing visual association areas V2 and V3 bilaterally, is part of the dorsal visual stream that projects to the posterior parietal cortex.^42^ Dorsal stream activity is associated with reaching and grasping behaviors guided by representations of the position, shape, and orientation of objects. Moreover, these visual association areas play a pivotal role in visual imagery.^43^ The inverse functional correlation between FTD atrophy and the dorsal occipital cortex suggests that FTD induces disinhibition of dorsal stream regions, which, in turn, may predispose some patients to engage in visual art early in the illness. Because only a minority of patients with FTD report intensified VAC, we hypothesize that this network rebalancing^44^ may manifest as VAC only when certain conditions, such as a latent visual artistic talent or a conducive environment, are met.

Structural covariance analysis is a between-patients network mapping technique, which has been used to reveal regions that subserve particular behavioral or cognitive functions.^45^ Patients with VAC-FTD, when compared with those with NVA-FTD, demonstrated greater structural covariance between dorsomedial occipital cortex and the left primary motor cortex around the representation of the right hand. In our view, there are 2 possible explanations for this novel observation. First, as dorsomedial occipital cortex hyperactivity drives visual creativity in an artistically predisposed brain operating within a conducive environment, plastic cortical remodeling may, over time, enhance the structural correlation between visual and motor areas, reflecting patients’ new preoccupation. This hypothesis is supported by previous studies showing training-induced gray matter volume increases in visual association^46^ and primary motor^47,48^ regions. Second, the higher structural correlation between the dorsomedial occipital cortex and other brain areas may reflect a lifelong trait that predisposes some patients with FTD to develop VAC. The accounts are not mutually exclusive.

The PET-based single case with increasing occipital glucose metabolism in parallel to the emergence of VAC may also imply that hyperactivation of the dorsal stream predisposes to visual artistic engagement; however, further investigations to address the specificity of this finding are warranted.

### Limitations

The current study design did not allow further characterization of permissive factors for VAC to manifest (eMethods 11 in Supplement 1), a topic that can now be addressed in future studies. The study is further limited by the small VAC-FTD sample, which, despite being the largest VAC-FTD cohort reported to date, may have precluded detection of additional VAC-relevant brain regions.

## Conclusions

This work leveraged multimodal neuroimaging data to generate a novel hypothesis about the mechanisms underlying the emergence of VAC in FTD. Our findings suggest that lesion-induced activation of dorsal visual association areas may predispose some patients to the emergence of VAC, a remarkable gain of function that occurs early in the illness. This phenomenon is associated with greater structural covariance between dorsomedial occipital cortex and the left primary motor cortex, around the representation of the right hand. Future longitudinal studies are needed to further examine the hypothesis generated by this study and to shed light on other enhanced capacities arising early in the course of neurodegeneration.

## Notes

Supplement 1. eMethods 1. Ascertainment methodology to VAC-FTD group eMethods 2. Inter-rater reliability analysis eMethods 3. Clinical and neuropsychological measures eMethods 4. MRI quality control eMethods 5. Structural imaging preprocessing eMethods 6. Creation of individual W-score maps (individual atrophy maps) eMethods 7. Functional MRI data preprocessing eMethods 8. ^18^F-labelled fluorodeoxyglucose positron emission tomography (FDG-PET) images acquisition and preprocessing eMethods 9. Generation of FDG-PET W-score maps eMethods 10. Considerations for seed region selection for structural covariance analysis eMethods 11. Attempts to delineate patient related factors predisposing to visual artistic creativity eTable 1. Structural T1-weighted image acquisition protocols by scanner eTable 2. Demographic characteristics of patient groups and 175 healthy controls used to derive individual atrophy network maps eTable 3. Characteristics of patients with emergence of visual artistic creativity and frontotemporal dementia eTable 4. Neuropathological diagnoses of patients with frontotemporal dementia and emergence of visual artistic creativity (n = 6/17) eTable 5. Demographic characteristics of NVA-FTD, a patient group matched also for MMSE eTable 6. Interaction models results eTable 7. Brain regions that increased in FDG-PET metabolism more than 0.5 W-scores in the patient scanned before and after onset of visual artistic creativity eFigure 1. Atrophy network mapping technique eFigure 2. Mean W-score maps: VAC-FTD, NVA-FTD eFigure 3. Frequency maps: VAC-FTD, NVA-FTD eFigure 4. Atrophy network mapping control analyses – controlling for seed size eFigure 5. Atrophy network mapping control analyses – different t thresholds eFigure 6. Structural covariance results in NVA-FTD* subgroup (matched also for MMSE, n = 34) eFigure 7. Overlap between individual atrophy network map, delta FDG-PET W-score map and occipital dorsomedial ROI eReferences

Supplement 2. Data sharing statement
