# Supplementary material for: Prevalence, Timing, and Network Localization of Emergent Visual Creativity in Frontotemporal Dementia
Source: JAMA Neurol. 2023 Feb 27;80(4):377–87. doi: 10.1001/jamaneurol.2023.0001 (PMC9972248; doi:10.1001/jamaneurol.2023.0001)
Supplement: Supplement 1. — eMethods 1. Ascertainment methodology to VAC-FTD group eMethods 2. Inter-rater reliability analysis eMethods 3. Clinical and neuropsychological measures eMethods 4. MRI quality control eMethods 5. Structural imaging preprocessing eMethods 6. Creation of individual W-score maps (individual atrophy maps) eMethods 7. Functional MRI data preprocessing eMethods 8. 18F-labelled fluorodeoxyglucose positron emission tomography (FDG-PET) images acquisition and preprocessing eMethods 9. Generation of FDG-PET W-score maps eMethods 10. Considerations for seed region selection for structural covariance analysis eMethods 11. Attempts to delineate patient related factors predisposing to visual artistic creativity eTable 1. Structural T1-weighted image acquisition protocols by scanner eTable 2. Demographic characteristics of patient groups and 175 healthy controls used to derive individual atrophy network maps eTable 3. Characteristics of patients with emergence of visual artistic creativity and frontotemporal dementia eTable 4. Neuropathological diagnoses of patients with frontotemporal dementia and emergence of visual artistic creativity (n = 6/17) eTable 5. Demographic characteristics of NVA-FTD, a patient group matched also for MMSE eTable 6. Interaction models results eTable 7. Brain regions that increased in FDG-PET metabolism more than 0.5 W-scores in the patient scanned before and after onset of visual artistic creativity eFigure 1. Atrophy network mapping technique eFigure 2. Mean W-score maps: VAC-FTD, NVA-FTD eFigure 3. Frequency maps: VAC-FTD, NVA-FTD eFigure 4. Atrophy network mapping control analyses – controlling for seed size eFigure 5. Atrophy network mapping control analyses – different t thresholds eFigure 6. Structural covariance results in NVA-FTD* subgroup (matched also for MMSE, n = 34) eFigure 7. Overlap between individual atrophy network map, delta FDG-PET W-score map and occipital dorsomedial ROI eReferences [file jamaneurol-e230001-s001.pdf]

Supplemental Online Content

Friedberg A, Pasquini L, Diggs R, et al. Prevalence, timing, and network localization of emergent visual creativity in frontotemporal dementia. *JAMA Neurol*. Published online February 27, 2023. doi:10.1001/jamaneurol.2023.0001

eMethods 1. Ascertainment methodology to VAC-FTD group

eMethods 2. Inter-rater reliability analysis

eMethods 3. Clinical and neuropsychological measures

eMethods 4. MRI quality control

eMethods 5. Structural imaging preprocessing

eMethods 6. Creation of individual W-score maps (individual atrophy maps)

eMethods 7. Functional MRI data preprocessing

eMethods 8. ^{18}F-labelled fluorodeoxyglucose positron emission tomography (FDG-PET) images acquisition and preprocessing

eMethods 9. Generation of FDG-PET W-score maps

eMethods 10. Considerations for seed region selection for structural covariance analysis

eMethods 11. Attempts to delineate patient related factors predisposing to visual artistic creativity

eTable 1. Structural T1-weighted image acquisition protocols by scanner

eTable 2. Demographic characteristics of patient groups and 175 healthy controls used to derive individual atrophy network maps

eTable 3. Characteristics of patients with emergence of visual artistic creativity and frontotemporal dementia

eTable 4. Neuropathological diagnoses of patients with frontotemporal dementia and emergence of visual artistic creativity (n = 6/17)

eTable 5. Demographic characteristics of NVA-FTD, a patient group matched also for MMSE

eTable 6. Interaction models results

eTable 7. Brain regions that increased in FDG-PET metabolism more than 0.5 W-scores in the patient scanned before and after onset of visual artistic creativity

eFigure 1. Atrophy network mapping technique

eFigure 2. Mean W-score maps: VAC-FTD, NVA-FTD

© 2023 Friedberg A et al. *JAMA Neurology.*

eFigure 3. Frequency maps: VAC-FTD, NVA-FTD

eFigure 4. Atrophy network mapping control analyses – controlling for seed size

eFigure 5. Atrophy network mapping control analyses – different t thresholds

eFigure 6. Structural covariance results in NVA-FTD* subgroup (matched also for MMSE, n = 34)

eFigure 7. Overlap between individual atrophy network map, delta FDG-PET W-score map and occipital dorsomedial ROI

eReferences

This supplemental material has been provided by the authors to give readers additional information about their work.

eMethods 1: Ascertainment methodology to VAC-FTD group

The patient research notes reviewed for this study were obtained in the prospective cohort study: "Frontotemporal Dementia: Genes, Images and Emotions" focused on patients with frontotemporal dementia. Prolonged semi-structured interviews conducted by clinicians from multiple disciplines are depicted in highly detailed research charts with special emphasis on patients' baseline personality and cognitive and behavioral changes across multiple domains. This deep clinical phenotyping enabled the generation of this unique sample.

Combining patients across a phenomenological spectrum (de novo visual artistic skills and/or increase in quantity of visual art produced and/or substantial change in artistic style) was based on previous literature delineating enhancements in VAC in FTD spectrum disorders[1]. Combining patients across a phenomenological spectrum has been successfully employed in previous studies interrogating the neural underpinnings of lesion-related complex and rare behaviors. For example, in a study delineating the network localization of patients with delusional misidentification syndromes, Darby et al. ascertained patients with either a delusional belief involving the sense of under-familiarity for a person (Capgras) or place (reduplicative amnesia), or the feeling of over-familiarity for a person (Fregoli) or place (reduplicative paramnesia)[2].

eMethods 2: Inter-rater reliability analysis

To ensure reproducibility of the VAC-FTD ascertainment procedures, we conducted an inter-rater reliability analysis. Two raters (KN and MK), each blinded to prior ascertainment by the main rater (AF), were provided with the research records of the patients included in VAC-FTD and NVA-FTD groups (n = 68 total). The raters were asked to determine whether the patients met the operational definition of emergence of VAC (YES/NO) as presented in the manuscript. Inter-rater agreement, AF versus MK, was 94.1%, Cohen's Kappa 0.84; Inter-rater agreement AF versus KN was 92.6%, Cohen's Kappa 0.81; Inter-rater agreement MK versus KN was 95.6%, Cohen's Kappa 0.88. Fleiss' Kappa evaluating agreement among all three raters was 0.84. These findings confirmed that the ascertainment of patients according to these criteria was reproducible.

eMethods 3: Clinical and neuropsychological measures

All neurological and neuropsychological assessments occurred within 180 days of MRI scanning that was used as index. The Neuropsychiatric Inventory Questionnaire (NPI-Q) was used to examine differences in neuropsychiatric behavioral alterations between VAC-FTD and NVA-FTD (table 1). The NPI-Q rates the existence, frequency, and severity of neuropsychiatric behavioral alterations within the last month across twelve domains[3]. Information with regards to frequency of abnormal behaviors across the twelve domains measured by the NPI-Q was not systematically collected in both VAC-FTD and NVA-FTD groups. We used the information with regards to the presence (YES/NO) of each abnormal behavior in between-group comparisons which was available for all participants and was less influenced by informant's characteristics (gender, cultural background etc.).

eMethods 4: MRI quality control

All MRI images were visually inspected by AF, IIG or LP. Additionally, we obtained MRI quality control measures using the CAT12 toolbox in SPM12 (running in MATLAB r2018b). Following these procedures all images with significant motion artifacts or lesions were excluded from further analyses.

eMethods 5: Structural imaging preprocessing

Structural images were segmented into gray matter, white matter, and cerebrospinal fluid and normalized to MNI space using SPM12 (http://www.fil.ion.ucl.ac.uk/spm/software/spm12/). Gray matter images were modulated by dividing the tissue probability values by the Jacobian of the warp field and smoothed with an isotropic Gaussian kernel with a full width at half maximum of 8 mm[4].

eMethods 6: Creation of individual W-score maps (individual atrophy maps)

A novel W-score model tailored to the characteristics of the study population was generated for this work, in keeping with prior publications[5,6]. Since VAC-FTD and NVA-FTD groups were ascertained from a cohort of patients with FTD spectrum disorders assembled over seventeen years, this model was designed to also account for the four different MRI scanners that used for image acquisition during the study period. Generation of the W-score model was finalized prior to neuroimaging analyses. To generate the W-score model[5,6], we first performed a voxel-wise general linear model (GLM) for the smoothed images of a cohort of 397 cognitively normal older adults

assessed at the UCSF Memory and Aging Center. Age at MRI, sex, handedness, years of education (YOE), scanner type (three distinct covariates, one for each scanner type) and total intracranial volume (TIV) were included as covariates. This sample had the following demographic characteristics: Mean age at MRI (standard deviation (SD)) = 69.3 (8.8) years; mean YOE (SD) = 17.2 (2.3) years; mean TIV (SD) = 1.4 (0.1) liters; male/female = 158/239; handedness right/left = 354/43; scanner 1.5T/3T/Prisma/4T = 58/144/140/52. While most demographical parameters were evenly distributed across sex and scanner type, an ANOVA model revealed significant differences in age across scanner type (F = 12.7; p<0.001). The smoothed images were subsequently used to derive an initial W-score model that included interaction terms for age and scanner type:

$$GM_{CON} = \beta0 + \beta1*Age + \beta2*Sex + \beta3*Handedness + \beta4*TIV + \beta5*Scanner\_1 + \beta6*Scanner\_2 + \beta7*Scanner\_3 + \beta9*YOE + \beta10*Scanner\_1*Age + \beta11*Scanner\_2*Age + \beta12*Scanner\_3*Age + \varepsilon$$

where GM_{CON} is the voxel-specific value of a segmented gray matter tissue density map in the control sample.
To estimate the statistical significance of each parameter coefficient map, corresponding t-maps were derived by dividing the parameter coefficient maps by the standard deviation of the residuals. These analyses revealed a negligible effect of scanner type*age interaction on expected gray matter intensity in our sample. Therefore, a final W-score model was estimated without the scanner type*age interaction terms:

$$GM_{CON} = \beta0 + \beta1*Age + \beta2*Sex + \beta3*Handedness + \beta4*TIV + \beta5*Scanner\_1 + \beta6*Scanner\_2 + \beta7*Scanner\_3 + \beta8*YOE + \varepsilon$$

Subsequently, individual W-score maps were computed for the patients as follows:

$$W = \frac{Observed - Expected}{SD\varepsilon}$$

Observed is the raw value of a voxel from the smoothed image of a patient; *Expected* is the expected value for the voxel of a specific patient adjusted for covariates using the healthy control model; and SDε is the standard deviation of the residuals from the healthy control model.

eMethods 7: Functional MRI data preprocessing

Functional MRI scans were processed using fMRIPrep[7] (RRID:SCR_016216). For anatomical image processing, the MPRAGE images were corrected for intensity non-uniformity (INU) with N4BiasFieldCorrection in ANTs [8], 2008) (RRID:SCR_004757), and used as T1w-reference throughout the workflow. The T1w-reference was skull-stripped with a Nipype[9] (RRID:SCR_002502) implementation of the antsBrainExtraction.sh workflow using OASIS30ANTs as target template. Brain tissue segmentation of cerebrospinal fluid (CSF), white-matter (WM) and gray-matter (GM) was performed on the brain-extracted T1w using FSL fast (https://fsl.fmrib.ox.ac.uk/fsl/fslwiki; RRID:SCR_002823). Volume-based spatial normalization to the MNI152NLin6Asym standard space was performed through nonlinear registration with antsRegistration, using brain-extracted versions of both T1w reference and the T1w template.
For functional image processing, the first five volumes were removed to allow for scanner equilibration. A reference volume and its skull-stripped version were generated by fMRIPrep. The BOLD reference was then co-registered to the T1w reference using FSL flirt with 6-degrees-of-freedom affine registration. Co-registration was configured with nine degrees of freedom to account for distortions remaining in the BOLD reference. Head-motion parameters with respect to the BOLD reference (transformation matrices, and six corresponding rotation and translation parameters) were estimated using FSL mcflirt and were used to compute the framewise displacement (FD). BOLD runs were slice-time corrected using AFNI 3dTshift (https://afni.nimh.nih.gov/; RRID:SCR_005927). The BOLD images were realigned from native to MNI152NLin6Asym standard space using antsApplyTransforms, configured with Lanczos interpolation, with a single interpolation step by composing transformations for head-motion and co-registrations to anatomical and output spaces. Images were spatially smoothed with a 6mm FWHM (full-width half-maximum) kernel using FSL susan. Confounding CSF and WM timeseries were calculated based on the preprocessed BOLD images, deriving average signals using the subject-specific anatomically derived tissue masks after erosion. The confound timeseries for head motion estimates, CSF, and WM were expanded with the inclusion of temporal derivatives and

© 2023 Friedberg A et al. *JAMA Neurology.*

quadratic terms for each[10]. Bandpass filtering in the frequency range 0.008-0.08 Hz was performed on the confound timeseries and BOLD images using fslmaths and AFNI 3dBandpass respectively. Confound timeseries were then regressed out of the BOLD images using fslglm. Subjects with greater than 0.55 mm mean FD were excluded from subsequent analysis[11].

eMethods 8: ^{18}F-labelled fluorodeoxyglucose positron emission tomography (FDG-PET) images acquisition and preprocessing

PET radiochemistry and acquisition

[18F]FDG was purchased from a commercial vendor (IBA Molecular). PET scans were performed at Lawrence Berkeley National Laboratory using a Siemens ECAT EXACT HR PET scanner in 3-dimensional acquisition mode. 30 minutes of dynamic FDG data were obtained. Ten-minute transmission scans for attenuation correction were obtained either immediately before or after each[18F]FDG scan. PET data were reconstructed using an ordered subset expectation maximization algorithm with weighted attenuation. Images were smoothed with a 4mm Gaussian kernel with scatter correction. All images were evaluated before analysis for patient motion and adequacy of statistical counts.

PET pre-processing and analysis

All image processing and analysis was performed in Statistical Parametric Mapping version 12 (SPM12; http://www.fil.ion.ucl.ac.uk/spm). Reference regions were defined in native MRI space for each subject using subcortical parcellations from FreeSurfer. FDG-PET frames were summed and standard uptake volume ratios (SUVR) were calculated by normalizing the summed FDG image to mean activity in the pons for each subject[12].

Spatial normalization

FDG data was co-registered to the subject's skull stripped T1-weighted MRI. To allow across-subject comparisons, each subject's T1-weighted MRI was normalized to MNI (Montreal Neurological Institute) space using the skull stripped ch2 template, and the derived normalization parameters were applied to the subject's co-registered FDG volumes. All normalized images were smoothed with a 12-mm Gaussian kernel.

Partial volume correction

In a post-hoc analysis, a two-compartmental partial-volume correction to all MRI scans was applied in order to correct PET data for atrophy [13]. The correction procedure involved convolving a binary brain mask (a sum of grey and white matter segmented images from the subject's T1-weighted MRI obtained from FreeSurfer, eroded by one voxel) with the point-spread function specific to the PET tomography along all axes. This provided a means for estimating the percentage of brain tissue emitting radioactivity at each voxel. The PET counts for each voxel were then adjusted based on the percentage of estimated brain matter[14].

eMethods 9: Generation of FDG-PET W-score maps

For these statistical maps, we used a W-score model generated based on FDG PET scans derived from 71 healthy controls who were included in the Berkeley Aging Cohort Study (BACS) and Neuroimaging in Frontotemporal Dementia (NIFD) study. Details on BACS inclusion criteria can be found in previous publications[15]. For up-to-date information on NIFD participation and protocol, please visit http://memory.ucsf.edu/research/studies/nifd. Demographic characteristics of 71 controls used to generate the model were the following: mean age at scan (SD) = 68(15), male/female = 32/39, handedness right/left = 67/4, mean years of education (SD) = 16.8(2), mean MMSE(SD) = 28.9 (1.1). In this model W-scores were adjusted for age, sex handedness and years of education.

eMethods 10: Considerations for seed region selection for structural covariance analysis

Several factors directed us to consider the potential relevance of the dorsomedial occipital region revealed by atrophy network mapping as potentially contributing to the emergence of VAC in FTD. First, the cluster size in 15/17 FTD-VAC and 45/51 NVA-FTD in the atrophy network maps thresholded at t>|7| was greater in VAC-FTD than in NVA-FTD (2394 and 1254 voxels respectively, figure 3 panels A and B). This cluster was present in 17/17 of VAC-FTD and 45/51 of NVA-FTD. Similar differences in prevalence and size were shown using different statistical thresholds (eFigure 4,5). Second, previous studies showed that diverse enhancements in visual behaviors occur in patients with FTD spectrum disorders. Viskontas et al. demonstrated that patients with svPPA showed more efficient visual search under distracting conditions than healthy controls[16]. Patients with svPPA were also found to have highly preserved and even enhanced capacity to solve jigsaw puzzles[17]. These findings suggest that lesion induced visual enhancement may not be specific to patients who engage in conventional forms of visual creativity and might manifest in several ways, which depend on additional patient characteristics. The lack of significant

© 2023 Friedberg A et al. *JAMA Neurology.*

between-group differences in the unthresholded t-maps does not preclude a possible dorsomedial occipital cortex contribution to emergence of VAC. Taken together these findings led us to hypothesize that dorsomedial occipital cortex enhancement may set the stage for VAC in some patients and led us to further explore this region in additional analyses.

Since visual creativity is a complex repetitive behavior involving multiple brain regions, we hypothesized that its neural signature would be reflected in long-standing interactions between relevant brain structures. Brain areas highly correlated in volume are often part of functional-anatomical systems known to subserve particular behavioral or cognitive functions. For example, posterior and anterior language areas in the left hemisphere co-vary strongly in cortical thickness[18]. Structural covariance analysis was one of the only feasible methods for interrogating neural networks in the patients because structural MRI was the only neuroimaging modality available for all VAC-FTD subjects.

eMethods 11: Attempts to delineate patient related factors predisposing to visual artistic creativity

We reviewed the research charts of VAC-FTD and NVA-FTD for (1) evidence of artistic talent before the age of eighteen (2) artistic background in each patient's family history (3) change in environment that may lead to visual artistic occupation (for example a family member enrolling the patient to an art class). Because information was not available for the majority of members in both VAC-FTD and NVA-FTD, conclusions could not be drawn. Future prospective data collection is needed to better understand predisposing factors to emergence of VAC.

© 2023 Friedberg A et al. *JAMA Neurology.*

eTable 1: Structural T1-weighted image acquisition protocols by scanner.

	**Scanner I**	**Scanner II**	**Scanner III**	**Scanner IV**
**Manufacturer (system)**	Siemens (Tim Trio)	Siemens (Magnetom Prisma)	Siemens (Magnetom VISION)	Siemens (Bruker MedSpec)
**Magnet strength**	3T	3T	1.5T	4T
**Repetition time (ms)**	2300	2500	5000	2300
**Echo time (ms)**	2.98	2.82	20	3
**Slice Thickness (mm)**	1	1	1.5	1
**Voxel size (mm)**	1 x 1 x 1	1 x 1 x 1	1.5 x 1.5 x 1.5	1 x 1 x 1
**Groups of scanned participants, VAC-FTD/ NVA-FTD/HC/HC-ATN**	3/11/51/175	5/24/0/0	7/13/0/0	2/3/0/0

Abbreviations: mm = millimeters; ms = milliseconds; VAC-FTD = patients with emergence of visual artistic creativity and frontotemporal dementia spectrum diseases, NVA-FTD = patients without emergence of visual artistic creativity and frontotemporal dementia spectrum diseases, HC=healthy controls, HC-ATN = healthy controls that were used for generation of individual atrophy network maps.

eTable 2: Demographic characteristics of patient groups and 175 healthy controls used to derive individual atrophy network maps

	**VAC-FTD**	**NVA-FTD**	**HC-ATN**	***P*-value**
n	17	51	175	
Age at MRI[a], years	65(9.7)	64.8(7)	65.0(8.6)	0.99
Male:Female[b], n	7:10	26:25	85:90	0.78
Right:Left[b], n	14:3	42:9	159:16	0.18
Education[c], years	16.1(4.7)	16.6(2.1)	17(2.2)	0.44

[a] One way analysis of variance (ANOVA)
[b] Chi square test
[c] Kruskal-Wallis test
Abbreviations: VAC-FTD = patients with emergence of visual artistic creativity and frontotemporal dementia spectrum diseases, NVA-FTD = patients without emergence of visual artistic creativity and frontotemporal dementia spectrum diseases, HC-ATN = healthy controls that were used for generation of individual atrophy network maps

© 2023 Friedberg A et al. *JAMA Neurology.*

eTable 3: Characteristics of patients with emergence of visual artistic creativity and frontotemporal dementia

Patient no.	Age at first FTD symptom	Age artistic skills emerge	Delta	Clinical diagnosis	Change in visual artistic creativity	Primary modality of visual art	Past artistic interest[a]
1	51-55	56-60	5	svPPA	Increase in quantity, change in style of visual art	Painting	++
2	41-45	56-60	16	bvFTD	De novo emergence of visual artistic creativity	Sculpting from tin objects	-
3	56-60	61-65	1	svPPA	De novo emergence of visual artistic creativity	Sculpture, Installation art	-
4	66-70	66-70	1	nfvPPA	De novo emergence of visual artistic creativity	Painting, making jewelry, glass painting	-
5	46-50	46-50	0	svPPA	Change in style	Painting	++
6	46-50	46-50	-1	svPPA	De novo emergence of visual artistic creativity	Painting, sculpture	-
7	51-55	51-55	3	svPPA	Change of modality from dance and music to verbal and visual art	Photography	+
8	41-45	46-50	2	bvFTD	De novo emergence of visual artistic creativity	Color pencil illustrations	-
9	46-50	56-60	9	bvFTD	Recrudescence of interest in visual art from his twenties	Painting, photography	+
10	61-65	31-35	-30	svPPA	Change of modality from dancing and opera singing to visual art	Pottery	+
11	56-60	61-65	3	PSP-RS	De novo emergence of visual artistic creativity	Sculpture in clay	-
12	56-60	41-45	-16	PSP-RS	Recrudescence of interest in visual art from her twenties	Quilting	+
13	66-70	71-75	3	svPPA	Reemergence of dormant artistic talent from childhood	Painting	+
14	56-60	51-55	-8	nfvPPA	Increase in quantity, change in style	Painting	+
15	81-85	86-90	1	CBS	Increase in quantity	Painting	+
16	56-60	56-60	2	svPPA	De novo emergence of visual artistic creativity	Creating montages with superimposed poems	-
17	56-60	56-60	-3	ALS	De novo emergence of visual artistic creativity	Making jewelry	+

[a] Past artistic interest: (++) Professional artist who experienced change in style (+) Visual art was a prior minor hobby and/or prior visual artistic education was obtained and /or there was any past interest in nonvisual art (-) No prior past artistic interest.

© 2023 Friedberg A et al. *JAMA Neurology.*

Abbreviations: svPPA = semantic variant of primary progressive aphasia, bvFTD = behavioral variant of frontotempotral dementia, nfvPPA = nonfluent variant of primary progressive aphasia, PSP-RS = progressive supranuclear palsy - Richardson syndrome, CBS, corticobasal syndrome, ALS amyotrophic lateral sclerosis.

© 2023 Friedberg A et al. *JAMA Neurology.*

eTable 4: Neuropathological diagnoses of patients with frontotemporal dementia and emergence of visual artistic creativity (n = 6/17)

Patient no.[a]	Primary neuropathological diagnosis	Secondary neuropathological diagnosis	ADNC level	LBD Stage
2	FTLD-TDP, Type B	Motor neuron disease, lower motor neuron only	Not ADNC	None
3	FTLD-tau, Pick's disease		Low	None
5	FTLD-TDP, Type C		Low	None
6	FTLD-TDP, Type A	Amyotrophic lateral sclerosis	Not to low	None
12	FTLD-tau, Corticobasal degeneration		Not ADNC	None
14	FTLD-tau, Corticobasal degeneration		Not to low	None

[a] Patient no. as in eTable 3. Abbreviations: FTLD=frontotemporal degeneration, ADNC=Alzheimer's disease neuropathological change, LBD= Lewy body disease, TDP= transactive response DNA-binding protein of 43 kDa

© 2023 Friedberg A et al. *JAMA Neurology.*

eTable 5: Demographic characteristics of NVA-FTD*, a patient group matched also for MMSE

	VAC-FTD (n=17)	NVA-FTD (n=34)	*P*
Clinical diagnosis : svPPA:bvFTD:nfvPPA: PSP-RS:CBS:ALS, n	8:3:2:2:1:1	16:6:4:4:2:2	
Age at MRI scan, years[a]	63.9 (61.1-70.36)	64 (60.5-69.75)	0.93
Male:Female, n[b]	7 : 10	20 : 14	0.372
Handedness (Right:Left)[b]	14 : 3	28 : 6	1
Education, years[c]	18 (16-20)	16 (16-18)	0.57
CDR-SB (max = 18)[a]	4.5 (2.5-6.5)	3.5 (2.125-6.375)	0.72
Mini-Mental State Exam (max=30)[c]	28 (27-29)	26 (24-28)	0.08

To address the question whether group level differences in structural covariance are driven by the difference in MMSE we generated an additional sample of 34 patients with frontotemporal dementia spectrum disorders without visual creativity (NVA-FTD*) who were matched to the VAC-FTD group for the previous parameters and MMSE. Median and interquartile range are reported. Results of subsequent neuroimaging analyses with this sample are shown in eFigure 6.

[a]Two-tailed two-sample t test

[b]Chi squared test

[c]Wilcoxon-Mann-Whitney test

Abbreviations: VAC-FTD = patients with emergence of visual artistic creativity and frontotemporal dementia spectrum diseases, NVA-FTD* = patients without emergence of visual artistic creativity and frontotemporal dementia spectrum diseases, matched also by MMSE, ROI = region of interest, R =right, MMSE = Mini Mental Status Examination.

© 2023 Friedberg A et al. *JAMA Neurology.*

eTable 6: Interaction models results

Model			x,y,z	T	Z	Region	BA
Interaction model VAC-FTD>NVA-FTD, MMSE as covariate of no interest							
	Cluster 1 (437 voxels)						
		Maximum 1	-54,-2,39	4.68	4.32	L premotor cortex	6
		Maximum 2	-52,-10,46	4.02	3.78	L primary motor cortex	4
		Maximum 3	-39,-3,60	4.01	3.77	L premotor cortex	6
	Cluster 2 (361 voxels)						
		Maximum 1	-58,-39,15	4.49	4.17	L superior temporal gyrus	22
		Maximum 2	-54,-30,9	3.7	3.51	L primary auditory cortex	41
		Maximum 3	-62,-24,10	3.67	3.48	L primary auditory cortex	41
Interaction model VAC-FTD>NVA-FTD							
	Cluster 1 (282 voxels)						
		Maximum 1	-54,-2,39	4.43	4.12	L premotor cortex	6
Interaction model VAC-FTD>HC							
	Cluster 1 (183 voxels)						
		Maximum 1	-53,-3,41	4.96	4.54	L premotor cortex	6
		Maximum 2	-41,2,50	3.88	3.67	L premotor cortex	6
	Cluster 2 (285 voxels)						
		Maximum 1	-45,29,26	4.86	4.46	L DLPFC	9
		Maximum 2	-45,24,33	4.8	4.42	L DLPFC	10
		Maximum 3	-41,11,32	3.87	3.66	L frontal eye field	8

© 2023 Friedberg A et al. *JAMA Neurology.*

	Cluster 3 (443 voxels)						
		Maximum 1	41,14,48	4.69	4.33	R Broca	44
		Maximum 2	45,15,33	4.13	3.87	R Broca	45
		Maximum 3	45,2,50	3.73	3.53	R premotor cortex	6
	Cluster 4 (290 voxels)						
		Maximum 1	18,35,48	4.68	4.32	R frontal eye field	8
		Maximum 2	14,44,41	4	3.76	R DLPFC	9
		Maximum 3	6,44,33	3.45	3.29	R dorsal ACC	32
	Cluster 5 (201 voxels)						
		Maximum 1	-47,-11,-39	4.54	4.21	L primary motor cortex	4
		Maximum 2	-53,-5,-24	3.73	3.54	L medial temporal gyrus	21
		Maximum 3	-53,-2,-33	3.7	3.51	L temporal pole	38
	Cluster 6 (326 voxels)						
		Maximum 1	62,-41,18	4.38	4.08	R superior temporal gyrus	22
		Maximum 2	56,-36,44	4.09	3.84	R supramarginal gyrus	40
		Maximum 3	57,-41,36	3.46	3.3	R supramarginal gyrus	41
	Cluster 7 (224 voxels)						
		Maximum 1	-30,-51,-51	4.23	3.95	L cerebellar hemisphere	NA
	Cluster 8 (189 voxels)						
		Maximum 1	44,-2,-41	3.98	3.75	R inferior temporal gyrus	20
		Maximum 2	48,-12,-38	3.85	3.64	R inferior temporal gyrus	21
		Maximum 3	48,5,-33	3.52	3.35	R temporal pole	38
	Cluster 9 (180 voxels)						

© 2023 Friedberg A et al. *JAMA Neurology.*

		Maximum 1	-50,24,9	3.98	3.75	L Broca area	45
		Maximum 2	-53,15,15	3.93	3.7	L Broca area	44

Abbreviations: VAC-FTD = patients with emergence of visual artistic creativity and frontotemporal dementia spectrum diseases, NVA-FTD = patients without emergence of visual artistic creativity and frontotemporal dementia spectrum diseases, HC = healthy controls, MMSE =mini mental status examination, BA= Broadmann area, R= right, L=left.

© 2023 Friedberg A et al. *JAMA Neurology.*

eTable 7: Brain regions that increased in FDG-PET metabolism more than 0.5 W-scores in the patient scanned before and after onset of visual artistic creativity

Brainnetome region number	Right/Left	Lobe	Gyrus	Delta
106	Right	Temporal lobe	Fusiform Gyrus	0.64
117	Left	Temporal lobe	Parahippocampal Gyrus	0.53
189	Left	Occipital Lobe	Medioventral Occipital Cortex	0.61
190	Right	Occipital Lobe	Medioventral Occipital Cortex	0.67
191	Left	Occipital Lobe	Medioventral Occipital Cortex	0.77
192	Right	Occipital Lobe	Medioventral Occipital Cortex	1.08
193	Left	Occipital Lobe	Medioventral Occipital Cortex	0.82
194	Right	Occipital Lobe	Medioventral Occipital Cortex	1.31
196	Right	Occipital Lobe	Medioventral Occipital Cortex	0.65
200	Right	Occipital Lobe	Lateral Occipital Cortex	0.51

© 2023 Friedberg A et al. *JAMA Neurology.*

eFigure 1: Atrophy network mapping technique.

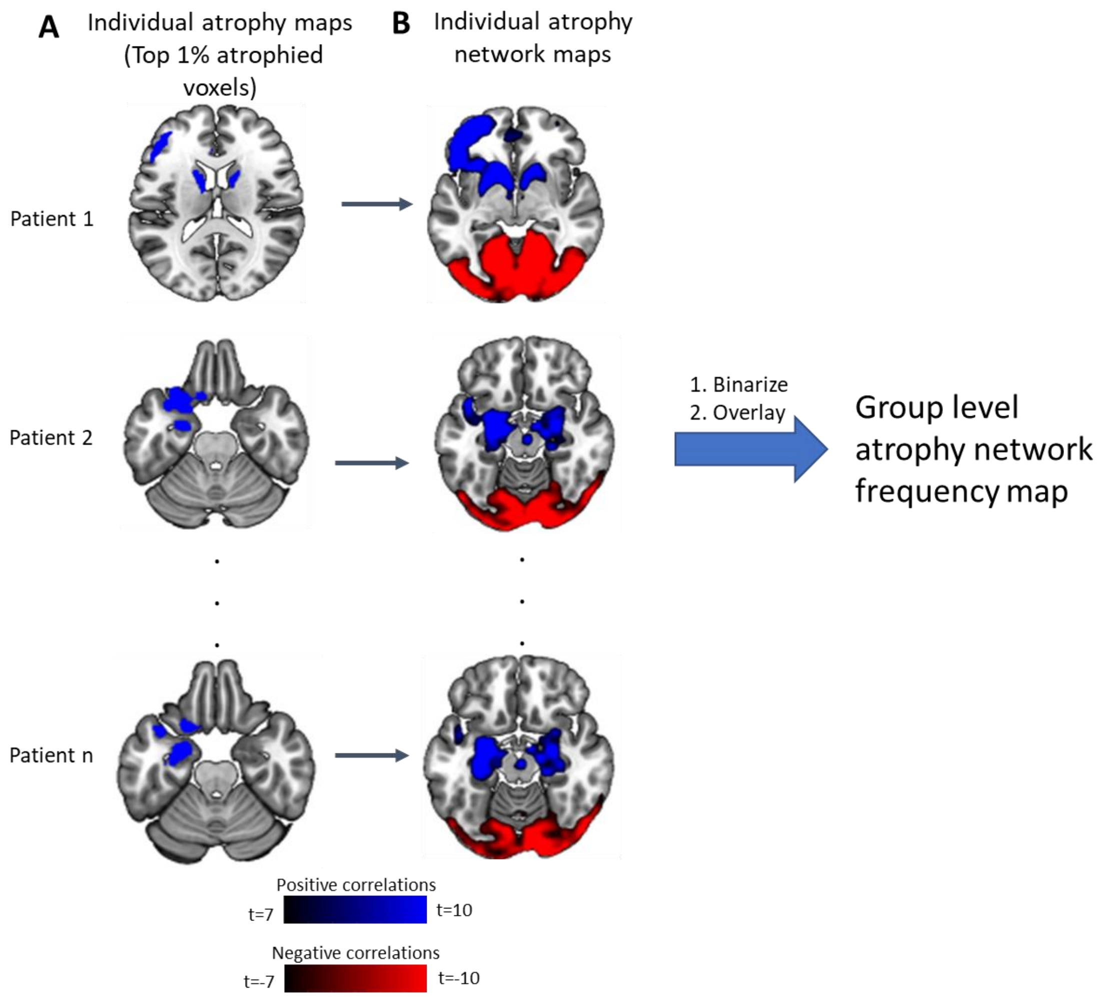

(A) Each patient's W-score map was binarized to generate individual atrophy maps (top 1% most atrophied voxels). (B) Regions positively and negatively functionally connected to each patient's atrophy map based on a connectome of matched healthy controls (n=175). Individual atrophy network maps of each group were thresholded, binarized and overlayed to generate group level atrophy network frequency maps.

© 2023 Friedberg A et al. *JAMA Neurology.*

eFigure 2: Mean W-score maps: VAC-FTD, NVA-FTD

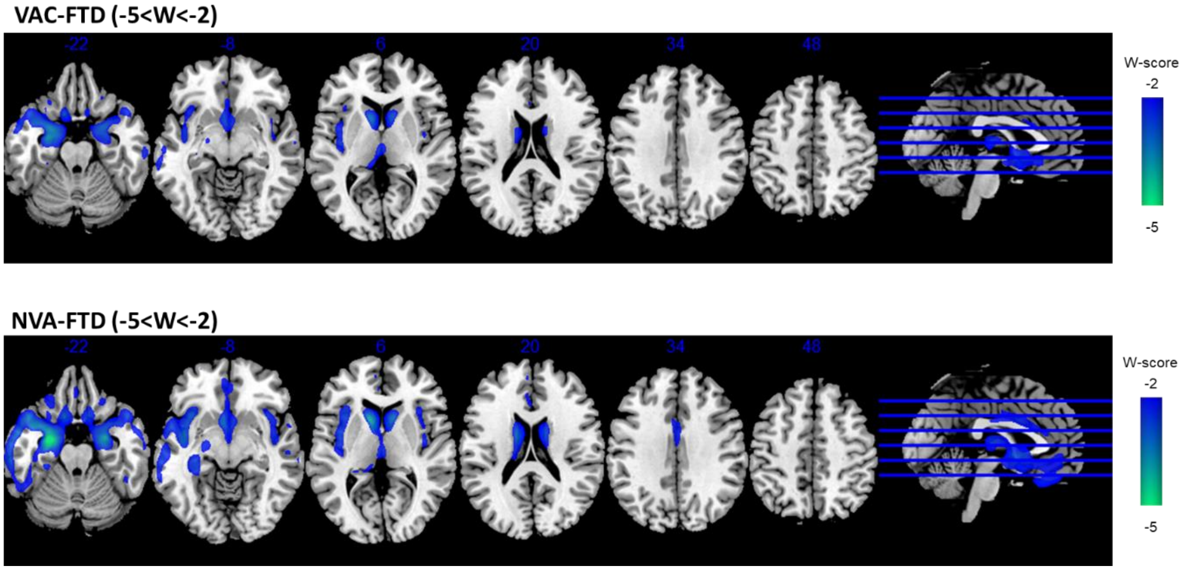

Findings are projected on Montreal Neurological Institute template brain. Images are in neurological orientation (left = left).
Abbreviations: VAC-FTD = patients with emergence of visual artistic creativity and frontotemporal dementia spectrum diseases, NVA-FTD = patients without emergence of visual artistic creativity and frontotemporal dementia spectrum diseases.

© 2023 Friedberg A et al. *JAMA Neurology.*

eFigure 3: Frequency maps: VAC-FTD, NVA-FTD

VAC-FTD

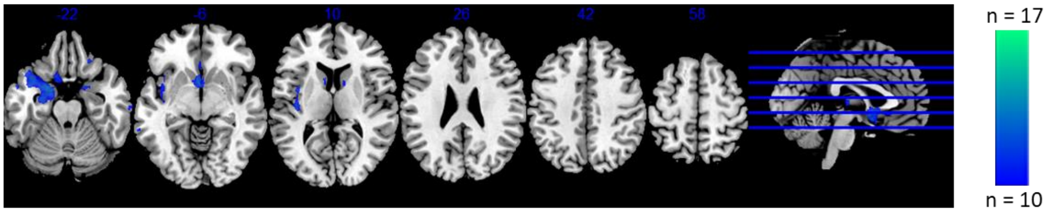

NVA-FTD

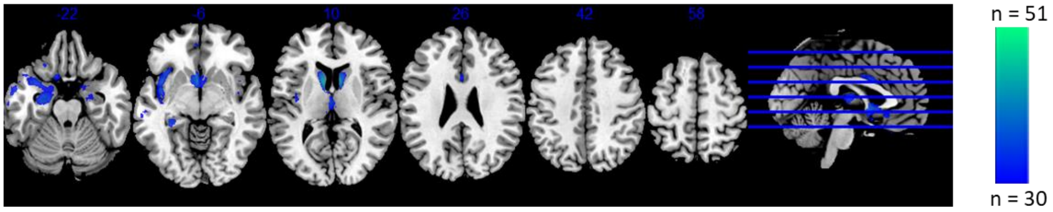

Individual W-score maps were thresholded at W<-2, binarized and overlayed to produce frequency maps for each group. No clusters were demonstrated above n=12/17 VAC-FTD patients. Findings are projected on Montreal Neurological Institute template brain. Images are in neurological orientation (left = left). Abbreviations: VAC-FTD = patients with emergence of visual artistic creativity and frontotemporal dementia spectrum diseases, NVA-FTD = patients without emergence of visual artistic creativity and frontotemporal dementia spectrum diseases.

© 2023 Friedberg A et al. *JAMA Neurology.*

eFigure 4: Atrophy network mapping control analyses – controlling for seed size

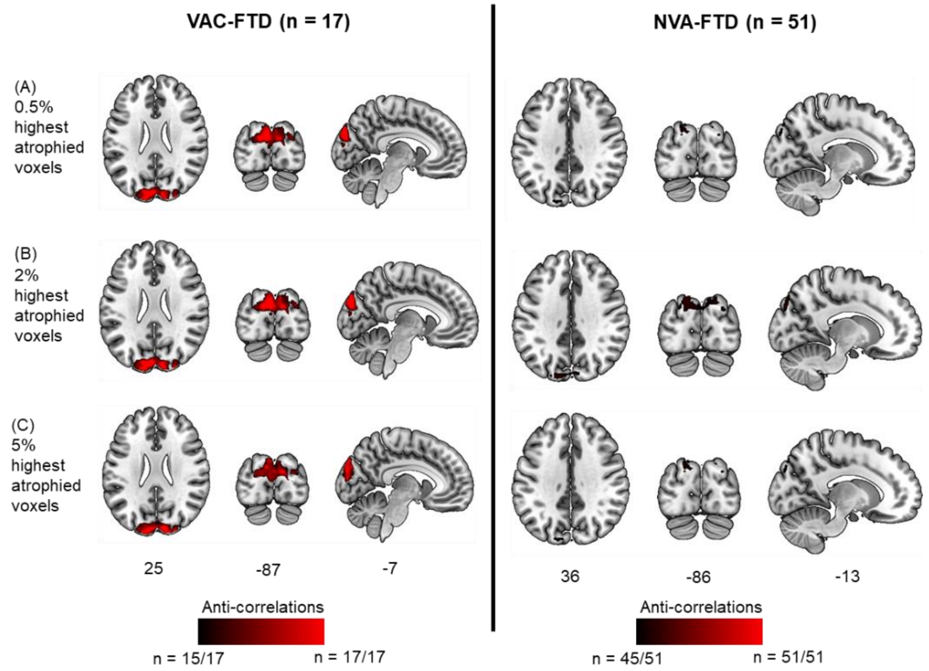

Group level atrophy network maps, generated by using seed regions-of-interest derived from the highest 0.5%,2% and 5% of atrophied voxels from individual patient atrophy maps, resulted in similar findings. Individual atrophy network maps were thresholded and binarized at |t| ≥7 and overlaid to produce group level atrophy network maps. No positive correlations to the atrophy patterns were detected using these thresholds. Findings are projected on Montreal Neurological Institute template brain. Images are in neurological orientation (left = left).

Abbreviations: VAC-FTD = patients with emergence of visual artistic creativity and frontotemporal dementia spectrum diseases, NVA-FTD = patients without emergence of visual artistic creativity and frontotemporal dementia spectrum diseases.

© 2023 Friedberg A et al. *JAMA Neurology.*

eFigure 5: Atrophy network mapping control analyses – different t thresholds

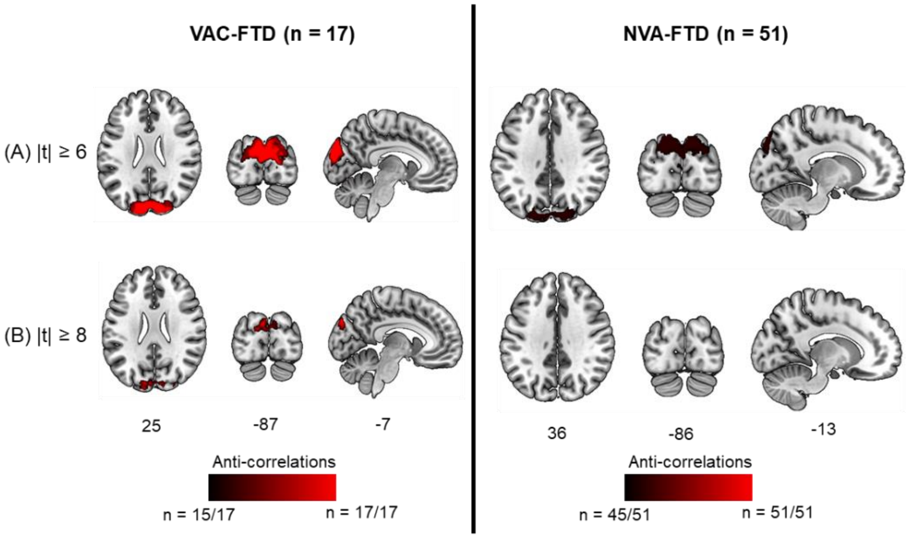

Individual atrophy network maps generated by seeding top 1% of atrophied voxels were thresholded at alternative statistical thresholds (|t|≥6, |t|≥8). Findings were overlaid to produce group level atrophy network frequency maps, which were similar those derived using |t|≥7. No positive correlations to the atrophy patterns were detected. Findings are projected on Montreal Neurological Institute template brain. Images are in neurological orientation (left = left).
Abbreviations: VAC-FTD = patients with emergence of visual artistic creativity and frontotemporal dementia spectrum diseases, NVA-FTD = patients without emergence of visual artistic creativity and frontotemporal dementia spectrum diseases.

© 2023 Friedberg A et al. *JAMA Neurology.*

eFigure 6: Structural covariance results in NVA-FTD* subgroup (matched also for MMSE, n = 34)

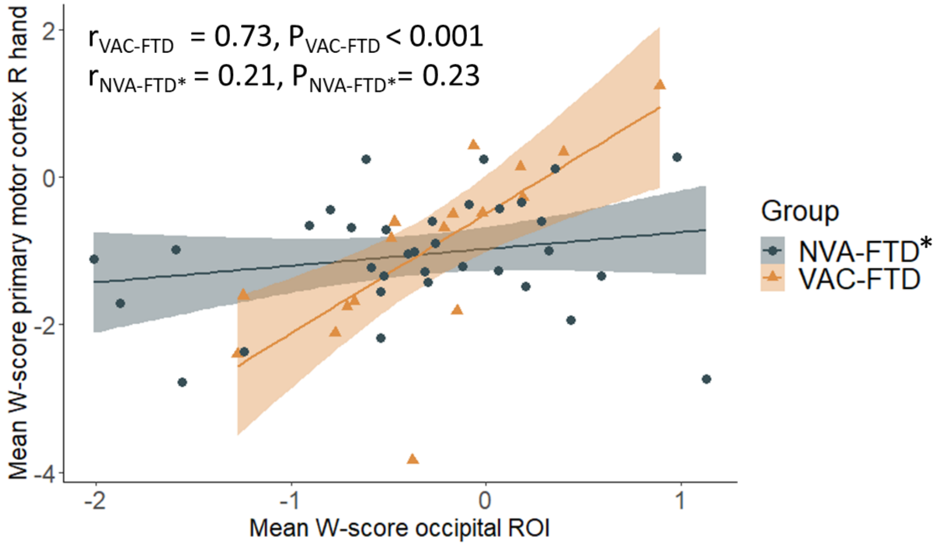

To address the question whether group level differences in structural covariance are be driven by the difference in MMSE we
To address the question whether group level differences in structural covariance are driven by the difference in MMSE, we generated an additional sample of 34 patients with frontotemporal dementia spectrum disorders without visual creativity (NVA-FTD*) who were matched for the previous parameters and MMSE (demographic characteristics are in eTable 5) to the VAC-FTD group. We then calculated the correlation in this group between the mean W-score of the dorsomedial occipital ROI is positively correlated with the mean W score in the right-hand region. No statistically significant correlation was found. In addition, we conducted a voxel-wise two sample t test comparing the W-score maps of VAC-FTD and NVA-FTD* and another two-sample t test unthresholded atrophy network maps. No statistically significant clusters were found using a statistical threshold of whole brain P_{unc}<0.001 (results not shown).
Abbreviations: VAC-FTD = patients with emergence of visual artistic creativity and frontotemporal dementia spectrum diseases, NVA-FTD* = patients without emergence of visual artistic creativity and frontotemporal dementia spectrum diseases, matched also by MMSE, ROI = region of interest, R =right, MMSE = Mini Mental Status Examination.

© 2023 Friedberg A et al. *JAMA Neurology.*

eFigure 7: Overlap between individual atrophy network map, delta FDG-PET W-score map and occipital dorsomedial ROI

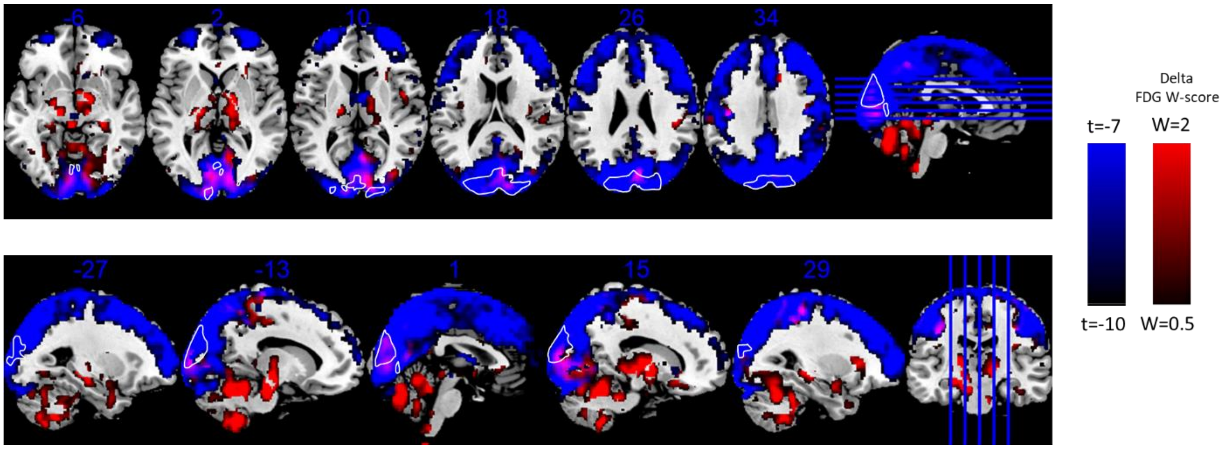

W-score maps derived from the FDG SUVR maps before and after emergence of VAC were subtracted to generate an individual delta map (red color scale) and thresholded at FDG W-score = 0.5 (positive values representing increase in metabolism). The patient's individual atrophy network map thresholded at t < -7 (regions anti-correlated to the seed) is shown in blue. Regions of overlap between the thresholded individual FDG W-score change map and the atrophy network map for the same subject are shown in purple. The group level occipital dorsomedial ROI found by atrophy network mapping is outlined in white. Areas of overlap (purple) within the white outlines are best seen in sagittal slices -13, 1, and 15 and indicate convergence of the single-subject data with the group-level findings. Images are in neurological view (left = left). Abbreviations: FDG-PET, [18F] fluorodeoxyglucose positron emission tomography; SUVR, standardized uptake value ratio; ROI, region of interest.

© 2023 Friedberg A et al. *JAMA Neurology.*

eReferences

1. Geser F, Jellinger KA, Fellner L, Wenning GK, Yilmazer-Hanke D, Haybaeck J. Emergent creativity in frontotemporal dementia. *J Neural Transm (Vienna).* 2021;128(3):279-293.
2. Darby RR, Laganiere S, Pascual-Leone A, Prasad S, Fox MD. Finding the imposter: brain connectivity of lesions causing delusional misidentifications. *Brain : a journal of neurology.* 2017;140(2):497-507.
3. Cummings JL, Mega M, Gray K, Rosenberg-Thompson S, Carusi DA, Gornbein J. The Neuropsychiatric Inventory: comprehensive assessment of psychopathology in dementia. *Neurology.* 1994;44(12):2308-2314.
4. Ashburner J, Friston KJ. Voxel-based morphometry--the methods. *Neuroimage.* 2000;11(6 Pt 1):805-821.
5. Jack CR, Jr., Petersen RC, Xu YC, et al. Medial temporal atrophy on MRI in normal aging and very mild Alzheimer's disease. *Neurology.* 1997;49(3):786-794.
6. La Joie R, Perrotin A, Barre L, et al. Region-specific hierarchy between atrophy, hypometabolism, and beta-amyloid (Abeta) load in Alzheimer's disease dementia. *J Neurosci.* 2012;32(46):16265-16273.
7. Esteban O, Markiewicz CJ, Blair RW, et al. fMRIPrep: a robust preprocessing pipeline for functional MRI. *Nat Methods.* 2019;16(1):111-116.
8. Avants BB, Epstein CL, Grossman M, Gee JC. Symmetric diffeomorphic image registration with cross-correlation: evaluating automated labeling of elderly and neurodegenerative brain. *Med Image Anal.* 2008;12(1):26-41.
9. Gorgolewski K, Burns CD, Madison C, et al. Nipype: a flexible, lightweight and extensible neuroimaging data processing framework in python. *Front Neuroinform.* 2011;5:13.
10. Satterthwaite TD, Elliott MA, Gerraty RT, et al. An improved framework for confound regression and filtering for control of motion artifact in the preprocessing of resting-state functional connectivity data. *Neuroimage.* 2013;64:240-256.
11. Parkes L, Fulcher B, Yucel M, Fornito A. An evaluation of the efficacy, reliability, and sensitivity of motion correction strategies for resting-state functional MRI. *Neuroimage.* 2018;171:415-436.
12. Minoshima S, Frey KA, Foster NL, Kuhl DE. Preserved pontine glucose metabolism in Alzheimer disease: a reference region for functional brain image (PET) analysis. *J Comput Assist Tomogr.* 1995;19(4):541-547.
13. Meltzer CC, Leal JP, Mayberg HS, Wagner HN, Jr., Frost JJ. Correction of PET data for partial volume effects in human cerebral cortex by MR imaging. *J Comput Assist Tomogr.* 1990;14(4):561-570.
14. Meltzer CC, Kinahan PE, Greer PJ, et al. Comparative evaluation of MR-based partial-volume correction schemes for PET. *J Nucl Med.* 1999;40(12):2053-2065.
15. Maass A, Landau S, Baker SL, et al. Comparison of multiple tau-PET measures as biomarkers in aging and Alzheimer's disease. *Neuroimage.* 2017;157:448-463.
16. Viskontas IV, Boxer AL, Fesenko J, et al. Visual search patterns in semantic dementia show paradoxical facilitation of binding processes. *Neuropsychologia.* 2011;49(3):468-478.
17. Green HA, Patterson K. Jigsaws-a preserved ability in semantic dementia. *Neuropsychologia.* 2009;47(2):569-576.

© 2023 Friedberg A et al. *JAMA Neurology.*

18. Alexander-Bloch A, Giedd JN, Bullmore E. Imaging structural co-variance between human brain regions. *Nat Rev Neurosci.* 2013;14(5):322-336.

© 2023 Friedberg A et al. *JAMA Neurology.*
