# Supplementary material for: Prevalence, Timing, and Network Localization of Emergent Visual Creativity in Frontotemporal Dementia
Source: JAMA Neurol. 2023 Feb 27;80(4):377–87. doi: 10.1001/jamaneurol.2023.0001 (PMC9972248; doi:10.1001/jamaneurol.2023.0001)
Supplement: Supplement 2. — Data sharing statement [file jamaneurol-e230001-s002.pdf]

Data Sharing Statement

Friedberg. Association of Emergent Visual Creativity in Frontotemporal Dementia With Brain Structure and Function. *JAMA Neurol*. Published February 27, 2023.
doi:10.1001/jamaneurol.2023.0001

Data
Data available: No

Additional Information
Explanation for why data not available: The datasets presented in this article are not readily available because of ethical and privacy restrictions. Requests to access the datasets should be directed to the corresponding author.
